# Prevalence of the metabolic syndrome in Luxembourg according to the Joint Interim Statement definition estimated from the ORISCAV-LUX study

**DOI:** 10.1186/1471-2458-11-4

**Published:** 2011-01-04

**Authors:** Ala'a Alkerwi, Anne-Françoise Donneau, Nicolas Sauvageot, Marie-Lise Lair, André Scheen, Adelin Albert, Michèle Guillaume

**Affiliations:** 1 Centre de Recherche Public Santé (CRP-Santé), Centre d'Etudes en Santé, Grand-Duchy of Luxembourg; 2 School of Public Health, University of Liège, Belgium; 3 Diabetes, Nutrition and Metabolic Disorders, Department of Medicine, CHU de Sart Tilman, Liege, Belgium

## Abstract

**Background:**

The prevalence of the metabolic syndrome (MS) has been determined in many countries worldwide but never in Luxembourg. This research aimed to 1) establish the gender- and age-specific prevalence of MS and its components in the general adult population of Luxembourg, according to the most recent Joint Interim Statement (JIS) definition, by using both the high and low cut-off points to define abdominal obesity, and 2) compare and assess the degree of agreement with the Revised National Cholesterol Education Programme-Adult Treatment Panel III (R-ATPIII) and the International Diabetes Federation (IDF) definitions.

**Methods:**

A representative stratified random sample of 1349 European subjects, aged 18-69 years, participated to ORISCAV-LUX survey. Logistic regression and odds ratios (OR) were used to study MS prevalence with respect to gender and age. The Framingham risk score (FRS) to predict the 10-year coronary heart disease (CHD) risk was calculated to compare the proportion of MS cases below or above 20%, according to both high and low waist circumference (WC) thresholds. Cohen's kappa coefficient (κ) was utilized to measure the degree of agreement between MS definitions.

**Results:**

The prevalence of the MS defined by the JIS was 28.0% and 24.7% when using the low (94/80) and the high (102/88) WC cut-off points, respectively. The prevalence was significantly higher in men than in women (OR = 2.6 and 2.3 for the low and high WC thresholds), as were all components of the MS except abdominal obesity measured by both thresholds. It also increased with age (OR values in age categories ranging from 2.7 to 28 when compared to the younger subjects for low WC and from 3.3 to 31 for the high WC cut-offs). The 10-year predicted risk of CHD by FRS did not depend on the threshold used. Globally, excellent agreement was observed between the three definitions of MS (κ= 0.89), in particular between JIS and IDF (κ = 0.93). Agreement was significantly higher in women than in men, and differed between age groups.

**Conclusion:**

Regardless of the definition used, the adult population of Luxembourg reveals a high MS prevalence. Our findings contribute to build evidence regarding the definitive construct of the MS, to help selecting the waist circumference thresholds for Europid populations, and to support the need to revise the guidelines for abdominal obesity levels.

## Background

The metabolic syndrome (MS) consists of a cluster of several metabolic and physiological abnormalities, including obesity, impaired glucose regulation, dyslipidemia and hypertension. It has become a subject of paramount interest in both research and clinical medicine, owing to its association with the increased risk of developing type 2 diabetes and atherosclerotic cardiovascular disease (CVD) [1-6]. During the last ten years, several working definitions have been proposed by various American, European and International organizations [7-12].

The multiplicity of definitions had an impact on the estimated MS prevalence rates in different populations and complicated the interpretation of epidemiological study results [13]. Several studies compared the concordance between the MS definitions which yielded a mixture of viewpoints regarding the most appropriate criteria [14], [15].

In addition, the recent guidelines released by the Joint Interim Statement (JIS) 2009[16] stressed the need to adopt ethnic-specific values of waist circumference (WC), to measure the central obesity criterion. For a given WC, Asians, Blacks, Caucasians showed different levels of intra-abdominal adiposity, hence putting the subjects at different risk levels of CVD and diabetes[17], [18].

So far, there is no agreement between the organizations, on the WC threshold to define abdominal obesity in people of European origin (Europid). While the IDF recommended a WC ≥ 94 cm for men and ≥ 80 cm for women, the European Cardiovascular Societies preferred cut points of ≥102 cm and ≥ 88 cm, respectively, for the two genders. Meanwhile, the JIS suggested using national or regional cut-off points for WC until more evidence from research work become available.

Despite the diversity of definitions, the prevalence of MS is well-known in various populations worldwide[19]. So far, however, there is no such information available in Grand-Duchy of Luxembourg. This work purposed for the first time to 1) establish the gender- and age-specific prevalence of MS and its components in the European adults residing in Luxembourg, according to the most recent JIS definition by using both the high and low WC cut-off values, and 2) compare the concordance with other operating definitions sharing substantially the same criteria (R-ATPIII and IDF).

## Methods

### Study population

A population-based, cross-sectional survey of cardiovascular risk factors (ORISCAV-LUX) was conducted in 2007-2008 in Grand-Duchy of Luxembourg. A representative random sample of 4496 non-institutionalized subjects residing in Luxembourg stratified according to gender, age (5-year categories) and geographic district (Luxembourg, Diekirch and Grevenmacher) was drawn from the regularly updated national health insurance registry. The distribution of selected subjects in each stratum was proportional to their distribution in the target population. A total of 1432 subjects took part in the survey, yielding a participation rate of 32.2%. A comprehensive description of the ORISCAV-LUX survey design, sampling method, and sample representativeness was published elsewhere [20], [21]. Briefly, selected persons were invited through an official letter followed by a phone contact to confirm the appointment. The participants were requested to keep at least 8 h fasting at attendance. The trained research nurses either visited participants in their households or invited them to the nearest study investigation centre. At the time of interview, the participants initially signed the informed consent form and then filled in the auto-administered questionnaire with the help of research staff. Given the multi-linguistic nature of the population residing in Luxembourg, the self-administered questionnaire was translated from French into the three other most used languages, namely German, English and Portuguese, and then backward translated into French to ensure the validity [22]. Information about past medical history, family history, demographic characteristics, socioeconomic status, physical activity, smoking, drinking and alimentary habits were collected. Subjects on regular medication were asked to bring their medicaments at attendance. The auto-administered questionnaires were chosen after careful review of the internationally available, tested and validated questionnaires on similar epidemiological studies.

Anthropometric measurements including height, body weight, as well as hip and WC, were conducted with the foot bare subjects wearing light clothing, by trained research nurses, according to a standardized operating procedure. Body weight (kg) was measured to the nearest 0.1 kg with electronic medical scales (Seca, Germany). Standing body height (cm) was measured to the nearest 0.2 cm with a portable wall stadiometer (Seca, Germany). Waist circumference (WC, cm) was measured at the level midway between 12^th ^rib and the uppermost lateral border of the iliac crest during mild expiration. WC was measured to the nearest 0.2 cm in standing position, using a flexible, non-distensible tape without exertion of pressure on the tissues. The body mass index (BMI) was calculated as weight divided by height squared (kg/m^2^).

Three consecutive measurements of sitting blood pressure at minimum 5-min interval were recorded, by using Omrom^® ^MX3 plus automated oscillometric Blood Pressure Monitor (O-HEM-742-E) (Matsusaka, Japan)[23], after the participants had been sitting for at least 30 minutes and refrained from smoking before the measurements. Systolic blood pressure (SBP, mmHg) and diastolic blood pressure (DBP, mmHg) were computed as the mean of the three measurements.

In addition, blood samples were collected from the antecubital vein to measure fasting plasma glucose (FPG, mg/dl), triglycerides (TG, mg/dl)), total cholesterol (TC, mg/dl), low-density lipoprotein cholesterol (LDL-C, mg/dl), high-density lipoprotein cholesterol (HDL-C, mg/dl), by using Roche (Switzerland) reagents on a P module of a Modular analyzer (Roche, Switzerland). All biochemical analyses were carried out within 2 h of blood sampling in the core laboratory of "Centre Hospitalier du Luxembourg (CHL)". The CHL laboratory applies strict internal and external standard quality control techniques.

### Definitions of metabolic syndrome

The last three operating definitions of MS were used. Participants were identified as having R-ATPIII MS[12] if they had three or more of the following criteria: 1) WC ≥ 102 cm for men and ≥ 88 cm for women; 2) raised concentration of TG ≥ 150 mg/dl or specific treatment for this lipid anomaly; 3) reduced concentration of HDL-C < 40 mg/dl for men and < 50 mg/dl for women or specific treatment for this lipid anomaly; 4) SBP was ≥ 130 mmHg, or DBP ≥ 85 mmHg or treatment of previously diagnosed hypertension; 5) FPG level ≥100 mg/dl or use of medication for hyperglycemia.

The IDF definition[8] requires central obesity as a prerequisite (WC ≥ 94 cm for men and ≥ 80 cm for women) plus two or more of the same remaining four criteria (raised concentration of TG, reduced concentration of HDL-C, elevated blood pressure and FPG level). Participants who self-reported a clinical diagnosis of diabetes by answering yes to the question "Did a doctor inform you that you have diabetes?", were considered as having this feature in the IDF-MS definition.

The JIS 2009 definition requires the presence of 3 out of the 5 above mentioned components to establish the diagnosis of MS. The dual references for Europid-specific WC cut-off points to define the abdominal obesity were applied; (1) WC ≥ 94 cm for men and ≥ 80 cm for women, as suggested by the IDF, and (2) WC ≥ 102 cm for men and ≥ 88 cm for women, as suggested by European Societies of cardiology. However, by applying the 102/88 thresholds, the definition criteria were exactly consistent with the R-ATPIII definition.

### Framingham risk score calculation

For every participant, the Framingham risk score (FRS) to predict 10-year coronary heart disease (CHD) risk was calculated using the adapted simplified model of Wilson et al [24], including the weighted risk factors: age, gender, blood pressure, LDL-C, HDL-C, smoking, and diabetes mellitus.

### Ethics

All participants were duly informed and consented to take part in the ORISCAV-LUX survey. The study design and collected data were approved by the National Research Ethics Committee and the National Commission for Private data Protection.

### Statistical analysis

Results were expressed as counts and proportions (%) for categorical variables. The overall prevalence of MS and of its individual components was estimated from the data and was associated with its 95% confidence interval (95%CI). Logistic regression analysis was applied to test the potential effect of age, gender and age-gender interaction on MS prevalence. Results were expressed in terms of the odds ratio (OR) together with its 95%CI.

To account for the stratified random sampling method, weighted statistical methods were applied. A sampling weight equal to the inverse probability of unit selection was allocated to each subject from the same stratum. This stratum sampling weight was defined as the ratio between the population stratum size and the observed sample stratum size.

The FRS was calculated for each individual and subsequently dichotomized to compare the proportion of MS cases with predicted 10-year risk of CHD below or above 20%, according to the two WC thresholds envisaged.

The percentage of subjects classified as MS was calculated for each pair of definitions as well as for the three definitions together. Finally, to measure the degree of agreement between MS definitions, Cohen's kappa coefficient (κ) was utilized: the closer κ to 1, the better the agreement between the definitions.

Results were considered to be significant at the 5% critical level (*P < 0.05*). All statistical analyses were performed by using the SAS 9.2 survey procedure for complex sampling design (© SAS Institute Inc., Cary, NC, USA).

## Results

### JIS definition based on higher and lower WC thresholds

A total of 1432 subjects participated in the ORISCAV-LUX survey. However, after eliminating the non-European residents and those with missing data on MS components, only 1349 participants were available for analysis.

Table 1 illustrates the JIS prevalence of MS and its components by using the two suggested WC cut-off values (102/88 and 94/80) among adult residents of Luxembourg. By applying the low thresholds, the MS prevalence was 28.0% as compared to 24.7% for the high thresholds. The prevalence of 102/88-based abdominal obesity criterion was markedly lower (30.6%) than that determined by the 94/80 cut-off points (51.7%). Among the other components, hypertension was the most commonly abnormal criterion (52.5%) in the study population.

**Table 1:** JIS-prevalence of the metabolic syndrome and of its components by using both high and low WC cut-off values (102/88 and 94/80) respectively among adults aged 18-69 years in the ORISCAV-LUX study

	n	JIS-94/80* (n = 1320)	JIS-102/88† (n = 1319)	Abdominal obesity 102/88 cut-off (n = 1348)	Abdominal obesity 94/80 cut-off (n = 1348)	Raised TG (n = 1320)	Low HDL-C (n = 1320)	High BP (n = 1348)	Hyperglycaemia (n = 1317)
Total	1349	28.0 (25.9 - 30.2)	24.7 (22.7 - 26.8)	30.6 (28.4 - 33.0)	51.7 (49.2 - 54.1)	25.6 (23.5 - 27.9)	18.8 (16.8 - 20.9)	52.5 (50.1 - 54.8)	21.7 (19.7 - 23.7)
**Gender**									
Women	692	20.4 (17.9 - 23.2)	18.5 (16.1 - 21.2)	35.1 (31.8 - 38.4)	57.7 (54.2 - 61.2)	14.7 (12.4 - 17.4)	16.2 (13.7 - 19.1)	42.2 (39.1 - 45.4)	14.4 (12.1 - 16.7)
Men	657	35.5 (32.2 - 38.9)	30.8 (27.6 - 34.0)	26.3 (23.3 - 29.6)	45.7 (42.2 - 49.2)	36.5 (33.0 - 40.2)	21.3 (18.5 - 24.6)	62.5 (59.0 - 65.9)	29 (25.8 - 32.2)
*P*-value		< 0.0001	< 0.0001	0.0002	< 0.0001	< 0.0001	0.014	< 0.0001	< 0.0001
**Age (years)**									
**Women**									
18-29	108	1.90 (0.5 - 7.4)	0.90 (0.1 - 6.4)	11.2 (6.5 - 18.6)	30.1 (22.6 - 38.9)	4.2 (1.6 - 10.2)	6.9 (3.3 - 14.0)	11.1 (6.3 - 18.7)	0.00 (-)
30-39	165	9.6 (5.8 - 15.5)	7.8 (4.5 - 13.1)	30.2 (23.6 - 37.8)	56.1 (48.0 - 63.8)	8.8 (5.3 - 14.4)	9.5 (5.7 - 15.5)	27.0 (20.4 - 34.7)	5.2 (2.7 - 9.9)
40-49	175	18.6 (13.4 - 25.2)	16.3 (11.4 - 22.6)	34.5 (27.6 - 42.1)	58.0 (50.4 - 65.2)	10.0 (6.2 - 15.7)	13.8 (9.2 - 20.2)	42.6 (35.6 - 49.8)	18.3 (13.2 - 24.9)
50-59	140	37.8 (30.2 - 46.2)	34.2 (26.8 - 42.4)	48.2 (40.3 - 56.2)	71.3 (63.4 - 78.1)	20.2 (14.3 - 27.7)	20.3 (14.3 - 28.1)	66.7 (58.6 - 73.9)	31.0 (24.0 - 39.0)
60-69	104	50.8 (41.0 - 60.6)	49.8 (40.0 - 59.7)	66.7 (57.4 - 75.8)	88.0 (80.2 - 92.9)	42.6 (32.2 - 52.6)	41.8 (32.5 - 51.7)	89.0 (80.6 - 94.0)	27.5 (19.8 - 36.7)
*P*-value		< 0.0001	< .0001	< 0.0001	< 0.0001	< 0.0001	< 0.0001	< 0.0001	< 0.0001
**Men**									
18-29	98	9.5 (5.1 - 17.1)	7.5 (3.6 - 15.0)	8.1 (4.1 - 15.4)	15.9 (10.2 - 23.9)	15.1 (9.1 - 24.2)	10.9 (5.8 - 19.6)	36.0 (27.5 - 45.6)	9.5 (5.0 - 17.4)
30-39	156	18.2 (12.9 - 25.1)	17.1 (12.0 - 23.9)	18.0 (12.8 - 24.7)	36.4 (29.3 - 44.1)	29.6 (22.9 - 37.3)	11.8 (7.6 - 17.9)	50.9 (43.2 - 58.6)	16.4 (11.3 - 23.2)
40-49	180	43.1 (35.9 - 50.5)	34.4 (27.6 - 41.8)	32.9 (26.4 - 40.1)	54.0 (46.6 - 61.2)	42.1 (34.9 - 49.7)	21.2 (15.8 - 27.7)	67.2 (60.1 - 73.7)	33.7 (27.2 - 41.0)
50-59	129	57.5 (48.6 - 66.0)	52.1 (43.2 - 60.8)	40.1 (31.9 - 49.0)	65.1 (56.2 - 73.0)	50.1 (41.5 - 58.7)	31.2 (23.9 - 39.7)	83.7 (76.1 - 89.2)	45.4 (36.9 - 54.1)
60-69	94	68.3 (57.8 - 77.3)	60.4 (50.0 - 70.0)	43.1 (33.9 - 52.7)	73.6 (63.2 - 81.9)	56.7 (46.4 - 66.5)	43.7 (33.7 - 54.2)	93.1 (85.5 - 96.8)	54.5 (44.0 - 64.6)
*P*-value		< 0.0001	< 0.0001	< 0.0001	< 0.0001	< 0.0001	< 0.0001	< 0.0001	< .0001

*JIS-MS based on WC ≥ 94 cm (men) and ≥ 80 cm (women) thresholds. † JIS-MS based on WC ≥ 102 cm (men) and ≥ 88 cm (women) thresholds, i.e. exactly consistent with the R-ATPIII criteria. Data are expressed as percentages (CI 95%). Non-fasting subjects were eliminated from the analysis.

The prevalence of MS was significantly higher in men than in women as measured by both WC thresholds (low; 35.5% vs 20.4%; *P *< 0.0001) and (high; 30.8% vs 18.5%, *P*< 0.0001), respectively. In terms of odds ratios, the risk of MS was increased by an age-adjusted factor 2.7 (95%CI: 1.7-3.0) in men for the low WC threshold, whereas for the high WC threshold, we found OR = 2.3 (95%CI: 1.7-3.0). Likewise, all MS components were significantly more common in men than in women except for abdominal obesity as measured by both WC thresholds. The waist threshold of 102 cm identified 26% of male subjects, while the 94 cm threshold identified 46% of them. In women, the threshold of 88 cm identified 35% of positive cases against 58% for the 80 cm threshold.

Regardless of the thresholds used, the MS prevalence increased remarkably with age in both genders. For the low WC threshold, when compared to the lowest age category (18-29 years), the gender-adjusted risk of MS increased by a factor 2.7 (95%CI: 1.4-5.4), 7.8 (95%CI: 4.1-15), 16 (95%CI: 8.6-31) and 28 (95%CI: 14-54) for subjects aged 30-39, 40-49, 50-59 and 60-69 years, respectively. For the high WC threshold, results were similar; when compared to the lowest age category (18-29 years), the gender-adjusted risk of MS increased by a factor 3.3 (95%CI: 1.5-7.2), 8.0 (95%CI: 3.8-17), 18 (95%CI: 8.5-39) and 31 (95%CI: 14-65) for subjects aged 30-39, 40-49, 50-59 and 60-69 years, respectively. Similar patterns were observed for all other components. No age-gender interaction was found for any of the compared definitions or individual components.

The prevalence of 94/80-defined central obesity was 88% in women and 74% in men aged 60-69 years. In contrast, the 102/88 thresholds classified 67% of women and 43% of men as having central obesity in the same age group.

### FRS and JIS definition

After excluding participants with history of CHD, subjects were cross-classified according to their MS status (MS or not MS) for each definition and to their 10-year predicted FRS (> 20% or < 20%). Regardless of the definition used, the proportion of subjects at high 10-year CHD risk was significantly greater in the MS group than in the non MS group. When focussing on the WC thresholds, the proportion of subjects at high CHD risk, by using the JIS-102/88 criterion, was comparable to that obtained with the JIS-94/80 definition [9.65% (95%CI: 6.50 - 12.8) against 8.55% (95%CI: 5.75 - 11.4)], respectively (Table 2).

**Table 2:** The JIS prevalence of metabolic syndrome according to 10-year predicted risk of coronary heart disease, using the two waist circumference thresholds

Metabolic syndrome	Total	10-year CHD risk (FRS ≥ 20%) n (%) (95% CI)	P-value†
**JIS-94/80**			
Yes	386	33 (8.55) (5.75 - 11.4)	< 0.0001
No	916	7 (0.76) (0.20 - 1.33)	
JIS-102/88*			
Yes	342	33 (9.65) (6.50 - 12.8)	< 0.0001
No	960	7 (0.73) (0.19 - 1.27)	

* JIS-94/80 means the MS as defined by the lower thresholds of the WC criterion (94 cm for men and 80 cm for women). JIS-102/88 means the MS as defined by the higher thresholds of WC criterion (102 cm for men and 88 cm for women). JIS-102/88 corresponds exactly to the R-ATPIII definition. † P value indicates the comparison between the 10-year CHD risk classes according to the presence of the MS for each WC threshold.

### JIS-94/80 versus IDF and R-ATPIII definitions

**Figure 1:**
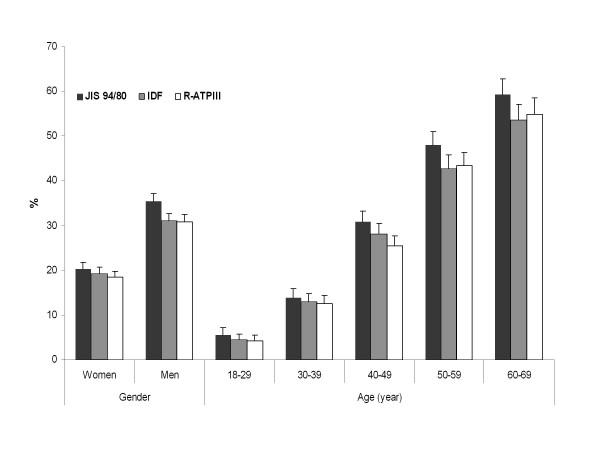
**Gender- and age-specific prevalence of the metabolic syndrome according to the R-ATPIII, IDF and JIS-94/80 definitions, among the adult residents of Luxembourg, ORISCAV-LUX study**.

To avoid confusion, only the JIS-94/80 will be used hereafter, as the JIS-102/88 corresponds exactly to the R-ATPIII. Figure 1 illustrates the gender- and age- specific prevalence of MS using the three definitions. Overall, JIS-94/80 yielded a higher MS prevalence than IDF and R-ATPIII in both genders. In men, the IDF and R-ATPIII prevalence rates were similar (31%), whereas the JIS-94/80 prevalence of the MS was notably higher (35.5%). In women, the MS prevalence rates by using JIS-94/80 (20.4%), IDF (19.3%) and R-ATPIII (18.5%) were quite comparable. For all age groups, the prevalence of MS by using the JIS-94/80 definition was higher than the IDF and R-ATPIII.

Table 3 reports the agreement between the 3 definitions according to gender and age groups. Among all participants, 97.2% were classified similarly under the JIS-94/80 and IDF definitions (κ = 0.93), likewise when comparing JIS-94/80 with R-ATPIII (96.7%, κ = 0.91). Lower agreement (93.9%, κ = 0.84) was found between the older definitions (IDF vs R-ATPIII). The degrees of agreement between the definitions were significantly higher in women than in men (*P*< 0.0001). Globally, agreement between the different age-specific prevalence rate estimates ranged from 0.69 to 0.96. Despite the general excellent concordance for the entire population, differences were noted between age groups.

**Table 3:** Percentage of observed agreements between the definitions of the metabolic syndrome among the adults aged 18-69 years residents in Luxembourg, ORISCAV-LUX study

Agreement* Percent ± SE (Cohen's kappa coefficient)				
	JIS-94/80 vs IDF	JIS94/80 vs R-ATPIII	IDF vs R-ATPIII	JIS-94/80 vs IDF vs R-ATPIII
Total	97.2 ± 0.46 (0.93)	96.7 ± 0.55 (0.91)	93.9 ± 0.65 (0.84)	93.9 ± 0.65 (0.89)
**Gender**				
Women	98.9 ± 0.38 (0.96)	98.1 ± 0.54 (0.94)	97.0 ± 0.65 (0.90)	97.0 ± 0.65 (0.94)
Men	95.5 ± 0.84 (0.90)	95.4 ± 0.80 (0.89)	90.8 ± 1.12 (0.78)	90.8 ± 1.12 (0.86)
*P*-value	0.0003	0.007	< 0.0001	< 0.0001
**Age (years)**				
18-29	98.8 ± 0.90 (0.89)	98.5 ± 0.84 (0.83)	97.3 ± 1.22 (0.69)	97.3 ± 1.22 (0.81)
30-39	99.0 ± 0.58 (0.96)	98.6 ± 0.72 (0.94)	97.6 ± 0.92 (0.90)	97.6 ± 0.92 (0.93)
40-49	97.3 ± 0.87 (0.94)	94.6 ± 1.18 (0.87)	91.9 ± 1.43 (0.80)	91.9 ± 1.43 (0.87)
50-59	94.7 ± 1.40 (0.89)	95.4 ± 1.31 (0.91)	90.1 ± 1.85 (0.80)	90.1 ± 1.85 (0.87)
60-69	94.3 ± 1.72 (0.89)	95.7 ± 1.55 (0.92)	89.9 ± 2.22 (0.81)	89.9 ± 2.22 (0.87)
*P*-value	0.0002	0.001	< 0.0001	< 0.0001

* Percentage of participants who were classified as either having or not having the metabolic syndrome under the considered definitions of the metabolic syndrome.

## Discussion

This work innovated by describing for the first time in Luxembourg the gender and age variation of the prevalence of MS and of its components in a representative sample of presumably healthy 18-69 years old adults, according to the most recent JIS definition. To evaluate the impact of different WC cut-off values for diagnosing MS, the prevalence rates were simultaneously estimated by using 102/88 and 94/80 thresholds, respectively. In addition, it aimed to compare and examine the concordance between the MS definitions currently in operation and sharing basically the same criteria.

Unsurprisingly, the prevalence of MS was higher by JIS-94/80 definition (28%) than JIS-102/88 definition (25%), a difference undoubtedly attributable to WC cut-offs. Similar findings were observed in some European countries but not in the United States where the difference in the MS prevalence, by using the high and low waist cut-off points, was relatively small because the prevalence of obesity in USA is higher whichever WC cut-off points used [16].

The emerging prevalence data during the current decade suggest that MS is quite prevalent worldwide, especially among older people, with gender-specific disparities. Consistently, in the ORISCAV-LUX study, the MS prevalence rates increased remarkably with age in both genders, as defined by both WC thresholds. This effect can be explained largely by age-related rises of blood pressure and glucose[25]. However, the significantly higher prevalence of MS and its components in men is of particular concern, especially for the younger age groups indicating their potential prolonged exposure to the proatherosclerotic risk factors associated with the MS.

Paradoxically, although the overall MS prevalence rate was significantly higher in men than in women, the prevalence of abdominal obesity was markedly higher in women, by using both high and low WC thresholds. This finding indicates that, in our Europid population, abdominal obesity was one but not the most important criterion, particularly in women. Other components such as elevated blood pressure and dyslipidemia may account more than or as much as abdominal obesity to determine the MS diagnosis, challenging therefore the appropriateness of the IDF criteria.

The suggested WC cut-off points to define the MS result from experts' consensus, thus call for validation by additional clinical and epidemiological prospective studies. While the WHO identified that a substantially high risk occurs at WC thresholds of (102/88)[26], the IDF and EGIR groups suggested lower values (94/80)[14]^, ^[27]. Waist circumferences of 102/88 thresholds were average values corresponding to a BMI of 30 kg/m^2 ^in men and women, respectively [28]. Within the context of the MS, the use of lower cutoff points is beneficial, as it raises the risk level for cardiometabolic disease among those identified as having the MS[29] and then indicates the need for early cardiovascular risk reduction. However, this approach implies a large impact on preventive strategies and health care resources. Our findings are important, not only to help decision-making with respect to the cardiovascular risk level intervention, but also to argument the future research work on the MS as a cardiometabolic outcome. As a proxy approach to prospective CHD risk prediction, the cross-sectional data of ORISCAV-LUX study were used to compare the proportion of MS cases with a 10-year predicted CHD risk exceeding 20%, according to both WC suggested thresholds. Our data suggest that both WC cut-off values would be appropriate as the predicted 10-year of CHD risk was similar.

Nevertheless, the established continuous relationship between WC and clinical outcomes makes the gender-specific values questionable, especially in women at menopause with an increase deposition of visceral fat [17]. Regardless of the thresholds applied, the consistent gender difference of 14 cm is discriminating, in the sense that a WC of 80 cm classifies women in a rather severe category, especially at the age of menopause where the women girth becomes physiologically larger. The ORISCAV-LUX findings demonstrated that more than 75% of women after the age of 50 years were classified as centrally obese by applying the 80 cm threshold. This issue suggests the need to reconsider the current IDF waist circumference recommendations, notably for the female elderly groups.

By comparing the three last definitions, the prevalence of JIS-94/80 MS was higher than IDF and R-ATPIII MS. This pattern of distribution was more prominent for men than for women. Regardless of the definition used, the prevalence of the MS increased across age groups.

Concerning the agreement between definitions, although the IDF placed more emphasis on central obesity in the causation of the MS, remarkable levels of agreement were found with the JIS (κ = 0.93) and good ones with the R-ATPIII (κ = 0.84), indicating that the requirement of abdominal obesity did not induce important discrepancies in the prevalence or the classification of the MS, but rather the WC cut-off points. Similar results were found in a large Chinese population [30] and other Caucasian populations[31]^, ^[32].

The use of different definitions has an impact on the estimated prevalence and confuses the interpretation of epidemiological studies[31]^, ^[33]^, ^[34]. A marked gender and American-European population's difference was observed[35]. In an attempt to harmonize the MS definition by comparing the R-ATPIII and the IDF criteria in American and German populations, Assmann et al. presumed that the observed prevalence discrepancy and particularly the gender-specific disagreement, by using the 2 different criteria, depend on the population characteristics, since a greater portion of German cohort population had lower WC measurements and were generally leaner than Americans[35]. Despite the intensive efforts of scientific societies to harmonize the criteria to define the MS, a key consideration remains controversial for WC cutoff points in the Europid population. Given the "almost perfect" level of agreement between the JIS- and R-ATPIII-defined MS (κ = 0.91), our results indicate that the two suggested cut-off points do not affect the prevalence estimates of the MS. In addition, the 10-year predicted CHD risk by FRS was similar for both thresholds-based definitions.

In general, a good agreement was found between the three definitions (κ = 0.89). This high degree of concordance and significant identification of a large number of participants as having the MS were not surprising, considering the fact that the three definitions use the same 5 components and that 4 out of five are defined identically [36]. In addition, despite the differences in their constructs, the concordance between the studied definitions was optimal in women as compared to men. In other words, the same individuals are essentially identified as having MS by using any construct criteria. In the most recent JIS definition, the IDF agreed to consider abdominal obesity as one of the 5 criteria and not deemed as a prerequisite element to diagnose the MS [16]. This harmonization is a step forward for a universal harmonization and allows a relevant international comparison.

The cross-sectional design of the ORISCAV-LUX study limits the possibility to determine which criteria better predicts adverse cardiovascular outcomes, such as the incidence of coronary events and thus precludes causal inferences. However, several strong points characterize the study. First, it is based on recent nationwide, population-based, representative sample of Luxembourg adult residents, from whom extensive direct measurements were obtained. A detailed study of non participants showed that the demographic and clinical characteristics of the ORISCAV-LUX participants were comparable with those of the non-participants[21]. The data were weighted to provide population-representative prevalence estimates.

In line with the 2009 JIS recommendation, a novel aspect of this study was to assess the MS according to the most recent definition together with the lower and higher criteria for Europids. Furthermore, the predominantly white homogenous nature of the sample (94.2% Europid) ensured the control over ethnicity factor, hence allowed generalizing the results.

## Conclusion

In conclusion, regardless of the MS definition, this representative sample of the Luxembourg Europid adult population demonstrated a high prevalence of MS, although using the IDF-defined abdominal obesity criterion (94/80) inflated the prevalence estimate, notably among elderly groups. This fact underscores the importance of promoting healthy lifestyles, such as proper nutrition, weight management, and adequate physical activity among the apparently healthy adults to fight against this emerging cardiometabolic disorder.

Our data contribute to build evidence regarding the definitive construct of the MS, to ascertain that both WC thresholds would be appropriate to define the prevalence of the MS in the Europid population, and to stress the need for revising the guidelines about the waist circumference cut-off points, particularly in women after menopause. These perspectives could be evidently accomplished by epidemiological longitudinal data.

## Abbreviations

CHD: Coronary heart disease; CVD: Cardiovascular disease; DBP: Diastolic blood pressure; EGIR: European Group for the Study of Insulin Resistance; FPG: Fasting Plasma Glucose; FRS: Framingham Risk Score; HDL-C: HDL cholesterol; IDF: International Diabetes Federation; JIS: Joint Interim statement; LDL-C: LDL cholesterol; MS: Metabolic syndrome; NCEP ATP III: National Cholesterol Education Program, Adult Treatment Panel III; ORISCAV-LUX: Observation of cardiovascular risk factors in Luxembourg; R-ATPIII: Revised National Cholesterol Education Programme-Adult Treatment Panel III; SBP: Systolic blood pressure; TC: Total cholesterol; TG: Triglycerides; WHO: World Health Organization.

## Competing interests

The authors declare that they have no competing interests.

## Authors' contributions

AAlk involved in the conception, design and coordination of the ORISCAV-LUX survey, involved in the present research design, contributed to statistical analysis and drafted the manuscript. A-FD performed the statistical analyses and contributed to the critical discussion of the results. NS participated in the statistical analysis. M-LL involved in the conception, design of the ORISCAV-LUX survey, and in the critical discussion of present results. AS and AAlb contributed to the critical revision of the manuscript and intellectual content. MG provided expertise and oversight throughout the process. All authors read and approved the final version.

## Pre-publication history

The pre-publication history for this paper can be accessed here:

http://www.biomedcentral.com/1471-2458/11/4/prepub
